# Natural compounds: Wnt pathway inhibitors with therapeutic potential in lung cancer

**DOI:** 10.3389/fphar.2023.1250893

**Published:** 2023-09-28

**Authors:** Xuetong Shen, Chundi Gao, Huayao Li, Cun Liu, Longyun Wang, Ye Li, Ruijuan Liu, Changgang Sun, Jing Zhuang

**Affiliations:** ^1^ College of First Clinical Medicine, Shandong University of Traditional Chinese Medicine, Jinan, China; ^2^ College of Traditional Chinese Medicine, Weifang Medical University, Weifang, China; ^3^ State Key Laboratory of Quality Research in Chinese Medicine and Faculty of Chinese Medicine, Macau University of Science and Technology, Taipa, China; ^4^ Department of Oncology, Weifang Traditional Chinese Hospital, Weifang, China

**Keywords:** Wnt/β-catenin signaling pathway, lung cancer, natural compounds, therapeutic potential, Wnt inhibitors

## Abstract

The Wnt/β-catenin pathway is abnormally activated in most lung cancer tissues and considered to be an accelerator of carcinogenesis and lung cancer progression, which is closely related to increased morbidity rates, malignant progression, and treatment resistance. Although targeting the canonical Wnt/β-catenin pathway shows significant potential for lung cancer therapy, it still faces challenges owing to its complexity, tumor heterogeneity and wide physiological activity. Therefore, it is necessary to elucidate the role of the abnormal activation of the Wnt/β-catenin pathway in lung cancer progression. Moreover, Wnt inhibitors used in lung cancer clinical trials are expected to break existing therapeutic patterns, although their adverse effects limit the treatment window. This is the first study to summarize the research progress on various compounds, including natural products and derivatives, that target the canonical Wnt pathway in lung cancer to develop safer and more targeted drugs or alternatives. Various natural products have been found to inhibit Wnt/β-catenin in various ways, such as through upstream and downstream intervention pathways, and have shown encouraging preclinical anti-tumor efficacy. Their diversity and low toxicity make them a popular research topic, laying the foundation for further combination therapies and drug development.

## 1 Introduction

With an estimated 2 million new diagnoses and 176 million deaths worldwide, lung cancer (LC) is the most prevalent malignancy and a major cause of cancer-related deaths (Thai et al., 2021). Its prognosis is one of the worst among all tumor types, with a 5-year survival rate of less than 20% (Allemani et al., 2018; Neal et al., 2019). Despite substantial advancements in disease management, the treatment of advanced LC is fraught with the risk of failure due to solid metastases, drug resistance, and the limited efficacy of current treatment options, including immunotherapy (Miller and Hanna, 2021). Therefore, there is an urgent need to identify new LC targets and develop safe treatment strategies.

The Wnt signal transduction cascade controls multiple embryonic and somatic processes, including cell fate classification, tissue repair, organ formation, and several pathological states (Nusse and Clevers, 2017). Notably, immoderate activation of Wnt signaling has been observed in numerous solid tumors, such as LC, and in other hematological tumors, such as leukemia. This abnormal activation can determine stem cell fate; promote tumorigenesis; and aid in tumor progression, metastatic spread, and drug resistance to chemotherapy, radiotherapy, immunotherapy, and targeting therapy (Ferrarelli, 2017; McMellen et al., 2020; Aguilera and Dawson, 2021; Yu et al., 2022).

Characteristic of adenocarcinomas, especially lung adenocarcinoma (LUAD), are abnormalities in the epigenetic control of the Wnt signaling system (Li et al., 2016a). Compared to normal tissues, the level of β-catenin in LC tissues was also significantly increased and was closely related to the differentiation and lymph node metastasis of LC. In addition, the recurrence rate in patients with high β-catenin expression was significantly higher than that in patients with low β-catenin expression (Liu et al., 2015). Wnt3a, a canonical Wnt pathway ligand, is overexpressed in 43% of squamous cell carcinomas and 51% of adenocarcinomas (Nardi et al., 2018). It was found that the expression of Wnt3a protein was low in normal or grade 1 LC tissues, but remarkably increased in higher-grade samples. Approximately 35%–70% of non-small cell lung cancers (NSCLC) are estimated to have anomalous Wnt signaling, depending on the quantification assay (Xu et al., 2011). Additional studies have confirmed that coercive activation of the Wnt pathway via engineered gene alleles promotes the advancement of KRAS- or BRAF-mutant lung adenomas (Juan et al., 2014; Rogers et al., 2018). The Wnt signaling cascade is a highly promising cancer target, and its diversity and importance provide great opportunities for the development of targeted therapies for LC (Tennis et al., 2007).

Targeted Wnt/β-catenin pathway therapy shows outstanding anti-LC efficacy, and a range of Wnt pathway inhibitors provide great potential for combination therapy in LC, according to preclinical studies conducted in recent years (Okada-Iwasaki et al., 2016; Scarborough et al., 2017). Unfortunately, most existing Wnt inhibitors have limited clinical application due to their non-negligible toxicity and side effects; thus far, no medicines that target this route have received FDA market approval (Krishnamurthy and Kurzrock, 2018; Torres et al., 2019) (Table 1). It is worth noting that acute inhibition of Wnt signaling or systemic elimination of Wnt secretion can affect intestinal homeostasis, leading to severe diarrhea, osteoblast and osteoclast differentiation, bone loss, and other serious side effects (Jung and Park, 2020). For example, the non-specific distribution of Wnt inhibitors leads to the development of diarrhea or fractures that force patients to discontinue treatment (Diamond et al., 2020). Therefore, a better understanding of the mechanism of this pathway will assist in new drug research to further explore new ways of targeting the Wnt/β-catenin pathway specifically or in combination. Therapeutic efficacy and toxicity should be perfectly balanced in treatments that target this route, given its extensive biological activity in LC.

**TABLE 1:** The Wnt pathway inhibitors in LC clinical trials.

Drug	Cancer type	Trial phase	Status	Clinical trial
LGK974	Lung Squamous Cell Cancer and some Malignancies Dependent on Wnt Ligands	I	Active, not recruiting	NCT1351103
OMP-54F28	Solid Tumors	I	Completed	NCT1608867
XNW7201	Advanced Solid Tumors	I	Completed	NCT3901950
CGX1321	Advanced Solid Tumors	I	Recruiting	NCT2675946
RXC004	cancer/Solid Tumors	I	Recruiting	NCT3447470
PRI-724	Advanced Solid Tumors	I	Terminated	NCT1302405
MCLA-158	NSCLC and Advanced/Metastatic Solid Tumors	I/II	Recruiting	NCT03526835
ETC-1922159	Solid Tumor	I	Recruiting	NCT02521844
DKN-01	Adenocarcinoma、Squamous cell carcinoma and other advanced solid tumors with Wnt-activated mutations	I/II	Available	NCT04681248
OMP-18R5	recurrent or advanced (Stage IV) NSCLC	I	Completed	NCT01957007
JS015	Advanced malignant solid tumors	I	Recruiting	CTR20223111
BI-905681	Advanced, unresectable and/or metastatic non-hematologic malignancies	I	Completed	NCT04147247

The data obtained from https://clinicaltrials.gov/ and https://db.pharnexcloud.com/. Last accessed on 30 April 2023.

The research prospects for plant-derived medicines are relatively broad owing to the variety of targets, good safety standards, and excellent synergism (Guan et al., 2022; Liu et al., 2022). Furthermore, sub-non-toxic-specific targeting may offer new therapeutic windows for LC therapy. To better understand the impact of the Wnt/β-catenin pathway on LC, we discuss its role in various aspects of tumor progression. With a limited number of potential Wnt inhibitors available in the clinic, this study is the first to systematically summarize the mechanism of action of flavonoids, polyphenols, alkaloids, terpenoids, and other natural products and their derivatives in the intervention of the Wnt signaling pathway in LC to meet the high demand for the development of more secure and manageable Wnt inhibitors. Various classes of compounds can interfere with multiple targets of the Wnt pathway to produce inhibitory effects and show great therapeutic potential when used alone or in combination with other drugs. Additionally, exploring these compounds in combination with existing anti-LC drugs could potentially enhance the effectiveness of current therapies and reduce the risk of drug resistance.

## 2 Composition and activation of the Wnt/β-catenin pathway

Wnt ligands are cysteine-rich proteins whose family consists of 19 secreted glycoproteins. Wnt ligand-mediated signaling pathways have a variety of physiological functions that have been conserved throughout evolution (Eubelen et al., 2018). Depending on whether they have a direct impact on β-catenin, the three pathways that the Wnt receptor activates are the canonical β-catenin cascade, the non-canonical planar cell polarity pathway, and the Wnt/Ca^2+^ pathway. These three pathways form a network of mutual regulation that finely coordinates various biological activities. Among them, the canonical pathway is closely related to the occurrence and development of LC and the most widely studied pathway; it is also the subject of this review.

**FIGURE 1:**
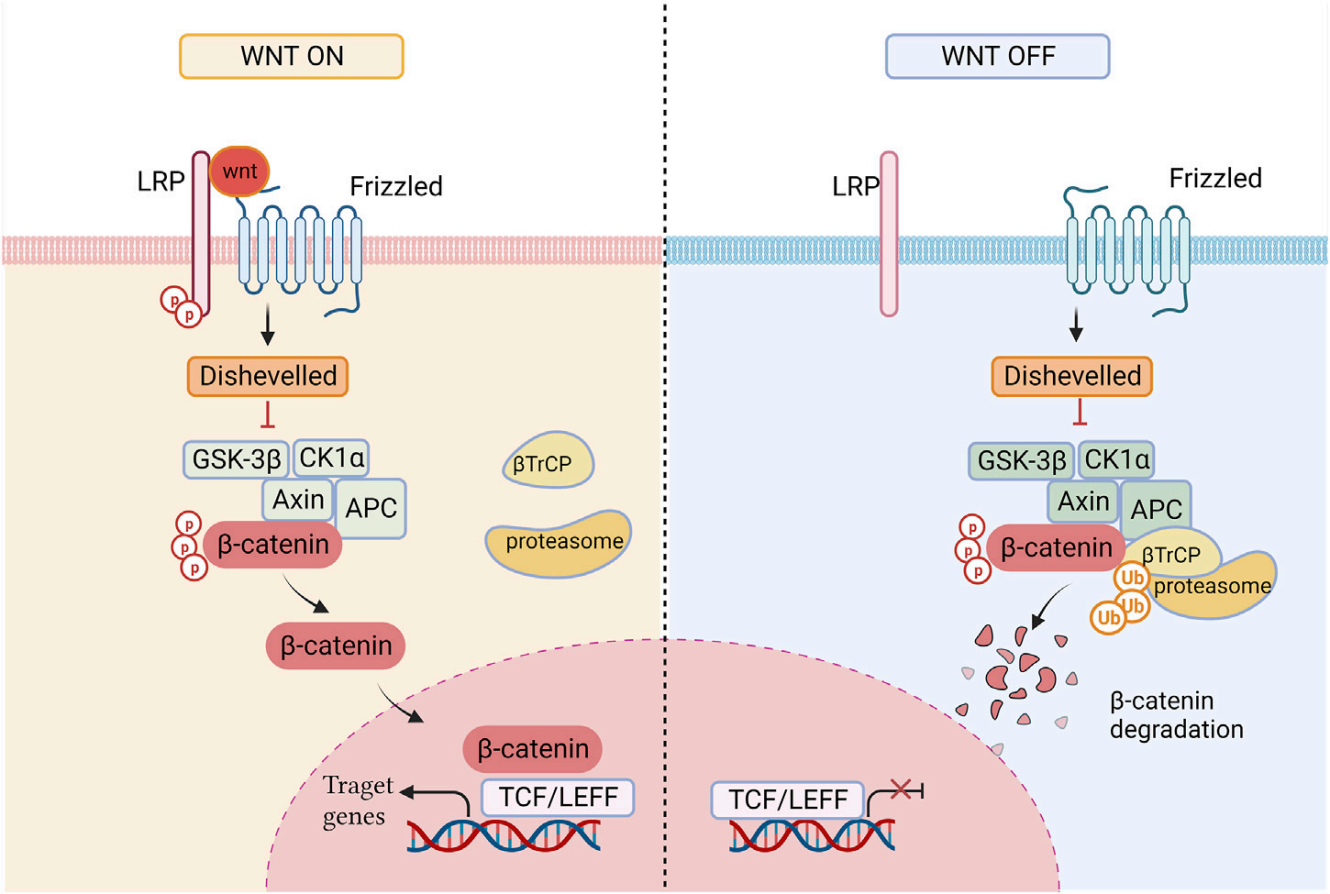
The state of the Wnt signaling path on and off. When Wnt ligand is present, the cytoplasmic concentration of β-catenin rises and β-catenin translocates to the nucleus, promoting the expression of target genes; In the Wnt Off state, β-catenin is degraded by ubiquitination.

The β-catenin-dependent pathway consists of three major components: transduction of Wnt signaling in the membrane, modulation of β-catenin stability in the cytoplasm, and overactivation of Wnt target genes in the nucleus (Nusse and Clevers, 2017). Ligand-related activation of the Wnt/β-catenin signaling pathway is induced by the binding of the Wnt ligand to the co-receptor complex, which includes the transmembrane domain protein frizzled (Fzd) and low-density lipoprotein receptor-related protein (LRP) 5/6. Activation of the Wnt pathway results in initiation intracellular signaling and membrane recruitment of Disheveled (Dsh/Dvl) which induces unsteadiness of the destruction complex made up of axis inhibition protein (Axin), glycogen synthase kinase-3beta (GSK-3β), casein kinase 1alpha (CK1α), and adenmatous polyposis coli (APC). This leads to the stabilization and accumulation of β-catenin in the cytoplasm and its subsequent nuclear localization. Β-catenin attaches to T-cell factors/lymphoid-enhancer factors (TCFs/LEFs) in the nucleus and elicits the transcription of Wnt target genes, such as c-myc (Myc), Cyclin D1 (CCND1), and survivin, to regulate cell proliferation, transdifferentiation, apoptosis, and other life processes (Yao et al., 2011; Okada-Iwasaki et al., 2016; Zhao H. et al., 2022). When no Wnt ligand is present, β-catenin is degraded by protein-destroying complexes. β-catenin is markedly degraded after phosphorylation by CK1α and GSK-3β. Dissociative β-catenin is then recognized by the E3 ubiquitin ligase-transducin repeat-containing protein (β-TrCP) and degraded via the ubiquitin-dependent proteasome pathway to maintain low levels of free β-catenin in the Wnt-off state (Stamos and Weis, 2013) (Figure 1).

There are various abnormal Wnt activation modes in malignant tumors, including acquired mutations, decreased expression of pathway inhibitors, and abnormal expression of ligands and receptors (Tortelote et al., 2017). Most sporadic colorectal tumors accumulate APC or β-catenin mutations, leading to abnormal Wnt signaling (Ferrarelli, 2017). Compared with colorectal cancer, activation of Wnt-mediated signaling takes place differently in LC, which is often associated with increased expression of Wnt pathway-activating effectors, such as Dvl and Wnt ligands, and downregulation of negative regulators, such as Axin and Wnt inhibitory factor-1 (WIF-1) (Mazieres et al., 2004; Mazieres et al., 2005; Tennis et al., 2007; Luo et al., 2018). It is clear that the mode of Wnt activation correlates with the type of tumor tissue. Therefore, future efforts to regulate this pathway more effectively may benefit from a clearer understanding of the unique aberrant activation of Wnt in LC.

## 3 Wnt/β-catenin in LC

Abnormally activated Wnt signalings frequently manifests in a variety of tumors that are widely involved in the occurrence and development of cancer. The Wnt signaling network controls a wide range of biological processes in LC (Katoh and Katoh, 2022). The undesirable activation of the Wnt/β-catenin signaling pathway has been linked to increased cancer prevalence, accelerated malignant progression, poor prognosis, and higher LC-related mortality (Xu et al., 2011; Parsons et al., 2021). Moreover, abnormal activation of the Wnt pathway interferes with stemness, proliferation, angiogenesis, metastasis/invasion treatment resistance, and other critical tumor developmental processes in LC, which are discussed below in light of the significant role of the classical Wnt pathway.

### 3.1 Maintain the stemness

Cancer stem cells (CSCs) are now considered the main “seeds” in tumorigenesis, development, metastasis and recurrence (Reya et al., 2001). Wnt signaling plays a role in the development of LCs by producing CSCs from non-CSCs or normal stem cells (Chaudhary et al., 2023). For example, the Wnt/β-catenin pathway is involved in the process of tobacco smoke (TS)-induced LC. After prolonged exposure to TS, human bronchial epithelial (HBE) cells are malignantly transformed and acquire CSC-like characteristics. In TS-transformed HBE spherical cells, silencing of β-catenin impeded the tumorsphere and reduced the expression levels of lung CSC markers (Wang J. et al., 2018). Similar to normal lung stem cells, CSCs enlist the Wnt, Notch, and Hedgehog signaling cascades to control stemness (Rowbotham et al., 2022).

Wnt/β-catenin signaling is thought to be necessary for maintaining the CSC phenotype. In CSCS, β-catenin dependent Wnt signaling is extremely active and contributes to maintaining the expression of putative lung CSC markers such as cluster of differentiation 44 (CD44) and cluster of differentiation 133 (CD133) (Teng et al., 2010; Zhu J. Y. et al., 2017). Compared to non-CSCs, LEF/TCF-binding elements have higher transcriptional activity, and Wnt signaling is increased in CSCs (Li X. et al., 2020). The high expression of Fzd 4/5 and higher sensitivity to Wnt3a-induced β-catenin dependent Wnt signaling further show that CSCs have heightened expression of Wnt downstream molecules compared with that in non-CSCs (He et al., 2021). In addition, transcription factor Stat3 is required for the proliferation and pluripotency of stem cells, and Stat3 activity is Wnt/β-catenin dependent, which suggests that Wnt signaling can control stem cell fate by affecting other pathways essential for stem cell maintenance (Peron et al., 2020).

Wnt signaling regulates complex biological behaviors that are beneficial for the survival and metastasis of lung CSCs. Through various target genes, Wnt pathway activation can aid CSCs in penetrating adjacent tissues and entering the bloodstream. During this time, tumor cells remain dormant, allowing them to survive and avoid immune monitoring through the adverse feedback of Wnt signaling. When CSCs reach appropriate sites, another burst of Wnt signaling induces successful CSC reinitiation and colonization of secondary organs (Malladi et al., 2016; Kim et al., 2017). In addition, a subset of p53-and KRAS-mutated lung carcinoma cells can serve as Wnt-producing niches for other cancer cell subsets. After treatment with the Wnt pathway inhibitor (LGK974), the lifespan of LC mice increased by nearly 50%, and leucine-rich repeat-containing G-protein-coupled receptor 5 (LGR5)+LUAD cells lost the potential for unlimited proliferation and metastasis similar to stem cells (Tammela et al., 2017). In summary, aberrant Wnt/β-catenin activation maintains LC stem cell-like characteristics and promotes LC progression. The Wnt/β-catenin signaling pathway has been implicated in the regulation of CSCs, although the underlying regulatory pathways of CSCs remain elusive (Cheng et al., 2023).

### 3.2 Participate in proliferation

Control of the cell cycle by Wnt signaling is responsible for the most mechanisms that promote proliferation during cancer (Pate et al., 2014). Wnt signaling is involved in LC cell cycle regulation in multiple mechanistic layers. Two key cell cycle regulators, Myc and CCND1, are direct target genes of the β-catenin/TCF transcription complex (Shtutman et al., 1999; Tetsu and McCormick, 1999). As one of the most important oncogenic transcription factors, Myc positively regulates multiple key cell cycle effectors including E2F transcription factors, cyclins, and cyclin-dependent kinases (Bretones et al., 2015). Wnt ligands accelerate tumor proliferation in NSCLC, partly by upregulating Myc and CCND1. The Ki-67 proliferation index of Myc-positive LC tissues is significantly higher than that of Myc-negative tissues (Huang et al., 2008). Compared to nearby normal tissues, CCND1 was significantly upregulated in NSCLC tissues and promoted cell proliferation and colony formation (Wu et al., 2020).

LC cells are also tightly regulated by multiple negative regulators and avoidance of these growth suppressors is necessary for tumorigenesis and facilitates cancer progression. Wnt signaling accelerates cell cycle progression by reducing the expression of many key cyclin-dependent kinase inhibitors (CDKNs) (Zhong et al., 2020). Nuclear accumulation of β-catenin in NSCLC is accompanied by increased cell proliferation. It was also observed that β-catenin expression was inversely correlated with CDKN1B (p27^KIP^), and dysregulation of β-catenin activity was shown to induce p53-dependent growth arrest in LC cells (Kotsinas et al., 2002). In addition, cancer cells acquire a characteristic resistance to apoptosis, which prevents them from dying and maintains uncontrolled proliferation (Jan and Chaudhry, 2019). Wnt3a triggers the canonical Wnt pathway to stop apoptosis caused by decreased levels of caspase-9 and caspase-3 (Wang T. L. et al., 2018). Suppressive Wnt/β-catenin signaling may encourage LC cell death and prevent proliferation through downstream transcription factors such as survivin, an inhibitor of the apoptosis family, although the precise mechanism is still unknown (You et al., 2004).

Tumor cell proliferation and malignant behavior require energy support (Patel and Powell, 2016) and CSCs prefer aerobic glycolytic energy metabolism, enabling constitutive proliferation and resilience to treatment-induced damage (Sancho et al., 2016). The Wnt pathway affects tumor cell energy metabolism through a variety of target genes (Pate et al., 2014). Myc is a major regulator of glycolysis (Dang et al., 2008). Myc-overexpressing LC cells exhibit enhanced aerobic glycolysis, whereas Myc-low cell lines appear to be more dependent on oxidative metabolism (Cargill et al., 2021). Activated Wnt/β-catenin signaling promotes Myc mRNA translation and expression and subsequently increases glycolysis and proliferation of LC cells (Yang X. et al., 2021; Huang et al., 2021).

### 3.3 Regulate angiogenesis

LC is a highly vascularized tumor and microvessel density (MVD) is the primary characteristic of intratumoral angiogenesis (Daum et al., 2020). Wnt signaling is an important factor in both pathological and physiological angiogenesis (Dejana, 2010). High-level angiogenesis is a crucial event in cancer cell dissemination and distant metastasis (Herbst et al., 2005), and hypoxia is considered a major catalyst for tumor angiogenesis (Brahimi-Horn et al., 2001). Hypoxia in LUAD cells can promote Wnt signaling by stabilizing β-catenin and altering its localization in the nucleus. Meanwhile, overexpression of hypoxia-inducible factors increases the content of β-catenin and enhances the resistance of LC cells to chronic hypoxia-induced stress (Hong et al., 2017). The most significant pro-angiogenic molecules, including members of the vascular endothelial growth factor (VEGF) family, matrix metalloproteinases (MMPs), and multiple chemokines, are regulated by canonical Wnt/β-catenin downstream signaling (Glaw et al., 2010). Numerous genes associated with tumor angiogenesis are expressed more frequently than usual when nuclear-β-catenin levels are elevated (Kasprzak, 2020). VEGF is a major mediator of the tumor microvasculature and is closely linked to the progression, metastasis, and recurrence of NSCLC. Previous studies have reported a positive correlation between β-catenin and VEGF protein expression in patients with NSCLC (Huang et al., 2008; Zhao Y. et al., 2022). In addition, tumor blood vessels are often leaky, poorly differentiated, and regardless of grade; one of the reasons for this phenomenon may be that differential Wnt expression induces modulation of cell morphology and function in LC (Rapp et al., 2017). For example, Wnt5a signaling can induce vasculogenic mimicry (Yao et al., 2014), while Wnt3a-induced Wnt activation is associated with increased angiogenesis (Shukla et al., 2016).

Wnt signaling can also indirectly affect angiogenesis by regulating the tumor microenvironment. For instance, angiogenesis can be induced by tumor-associated macrophages (TAMs) in the tumor microenvironment, which is thought to be the “switch” that first causes tumor angiogenesis (Cassetta and Pollard, 2023). TAMs release a range of pro-angiogenic substances that eventually lead to tumor angiogenesis due to the abnormally active Wnt pathway and autocrine signaling, which simultaneously promote M2 polarization (Cui et al., 2022). It is worth noting that although antiangiogenic therapy brings sufficient benefits to patients, its efficacy of antiangiogenic therapy is limited and is often accompanied by local hypoxia, tumor adaptation, progression, and metastasis (Uribesalgo et al., 2019). Studies have observed that when VEGF is inhibited, feedback activates Wnt signaling to increase the expression of β-catenin and CCND1, leading to a certain degree of upregulation of the proliferation and invasion abilities of human NSCLC A549 cells (Zhang X. X. et al., 2016). This implies that Wnt inhibitors can improve LC treatment when combined with antiangiogenic therapy.

### 3.4 Related to metastasis/invasion

Metastasis and invasion of the primary tumor and systemic tumor dissemination are the most deleterious events in the development of cancer, which pose challenging therapeutic difficulties and are significant contributors to cancer-related mortality (Hanahan and Weinberg, 2011). Changes that occur during LC initiation and metastasis can be described as epithelial-to-mesenchymal transition (EMT) (Brabletz et al., 2018). EMT-progressing cells exhibit altered phenotypes, lose epithelial features, and express more mesenchymal markers. The activation of EMT during cancer growth enables cancer cells to develop migratory, invasive, and stem cell-like characteristics (Liao and Yang, 2017; O'Leary et al., 2018). It has been demonstrated that Wnt signaling is essential for EMT and that the Wnt/GSK-3β axis regulates the degradation of Snail, an important mediator of EMT (Yook et al., 2005). Wnt signaling also regulates cell adhesion, triggering the adhesion junction protein E-cadherin, and upregulates the expression of the mesenchym-specific marker N-cadherin during EMT (Wu et al., 2017). Transforming growth factor-β (TGF-β), a key element in stimulating the EMT process, upregulated the expression of β-catenin, and the progression of EMT was positively correlated with the activity of Wnt/β-catenin pathway (Luan et al., 2022). Activation of Wnt/β-catenin signaling can also contribute to EMT induction either directly or indirectly by stimulating several EMT-related transcription factors, such as Slug, Twist, ZEB1, ZEB2, and E47 (Shin et al., 2010; Sánchez-Tilló et al., 2011). Notably, Wnt may also affect EMT by affecting CSCs, because the expression of the CSC marker CD44 is associated with the mesenchymal phenotype, suggesting a role in EMT induction and maintenance in LC (Suda et al., 2018).

Cell-cell adhesion and matrix depletion are important steps in the progression of all cancers from localized malignancy to metastatic disease. Wnt/β-catenin and its downstream products may affect the actin cytoskeleton and destabilize adhesion molecule junctions (Liu et al., 2023). Activation of Wnt/β-catenin signaling can work in conjunction with other oncogenic pathways such as KRAS in lung epithelial cells to produce a more aggressive cancer phenotype by forcing an embryonic distal progenitor phenotype and reducing E-cadherin expression (Pacheco-Pinedo et al., 2011; Hung et al., 2020). Moreover, MMPs enzymes directly regulated by Wnt can promote cancer metastasis by aiding the breakdown of the extracellular matrix and enabling cancer cells to spread throughout the lymphatic and blood arteries of the body (Ku et al., 2015). Wnt signaling may influence the organotropism of LC cells that progress along the metastatic cascade. A metastatic subpopulation isolated from node-derived lung adenocarcinoma cell lines has previously been shown to exhibit highly active Wnt/TCF signaling, and the Wnt/TCF target genes LEF1 and HOXB9 have been shown to promote LUAD cell colonization in the bone and brain (Nguyen et al., 2009). In addition, activation of the Wnt pathway in tumors results in a noninflammatory environment and suppresses immune surveillance through a variety of mechanisms (Du et al., 2023). This suggests that Wnt signaling may indirectly promote tumor growth and metastasis by inhibiting the immune-resistant tumor microenvironment.

### 3.5 Implicate in medication resistance

Systemic anti-LC treatments face great challenges, and drug resistance is primarily responsible for cancer patient mortality, whether directly or indirectly. The persistence of CSCs, enhanced multidrug resistance (MDR) and the downregulation of apoptosis are important causes of treatment failure (Yuan et al., 2017; Aleksakhina et al., 2019). It has been demonstrated that CSCs are more resistant to standard chemotherapy and radiotherapy. This may be because these cells produce large levels of anti-apoptotic proteins such as Myc, express more genes for drug resistance, and have more effective DNA damage repair (Ayob and Ramasamy, 2018). Several cancers, including LC, exhibit treatment resistance, given that Wnt signaling is crucial for sustaining CSCs (Yang et al., 2016b). Silencing β-catenin abolished Oct4/Nanog-mediated MDR and EMT in NSCLC cells (Liu et al., 2020).

MDR is the primary contributor to cancer chemotherapy failure (Wang et al., 2021). Wnt signaling activation is an important mechanism of chemoresistance in recurrent LC. Activation of Wnt signaling by APC inhibition resulted in chemoresistance in chemosensitive human LC cell lines, and higher Wnt activity was observed *in vitro* derived chemoresistant cell lines (Wagner et al., 2018). Overexpression of MDR-associated transporters is the key mechanism by which the aberrant activation of Wnt/β-catenin signaling results in the induction of cancer MDR (Ghandadi et al., 2019). The level of Wnt/β-catenin activity is positively correlated with resistance to several common chemotherapeutic drugs, such as paclitaxel and cisplatin, and radiation used in the treatment of LC (Yang et al., 2016b; Datta et al., 2019; Li et al., 2022). Frequently used adjuvant chemotherapy agents activate β-catenin-dependent signaling, which results in the upregulation of the expression and activity of multidrug-resistant protein 1 and other multidrug efflux transporters (Vesel et al., 2017). Upregulation of Wnt signaling protects cancer cells from cell cycle arrest or apoptosis which can promote tumor chemoresistance (Yuan et al., 2020). For example, the upregulation of β-catenin signaling may promote cisplatin resistance in LUAD cells through the overexpression of the BCL-XL gene (Zhang J. et al., 2016). Experiments have revealed that blocking Wnt signaling increases the radiosensitivity of LC cells (Wu et al., 2016; Li et al., 2022). Although this protective effect is attributed to downstream effectors of Wnt/β-catenin signaling, the more general mechanism of stress remains unclear (Lee et al., 2013).

Targeted therapy for LC is drug-resistant, partly because of the Wnt pathway. Adenocarcinoma is usually associated with activating mutations in the epidermal growth factor receptor (EGFR) gene and LC patients with activating mutations in the kinase domain of EGFR can be treated with tyrosine kinase inhibitors (TKIs) (Casás-Selves et al., 2012; Nakayama et al., 2014). Cross-regulation of EGFR and Wnt signaling in cancer results in abnormal increases in signaling proteins and transcription factors, leading to the failure of targeted therapies (Tabasum and Singh, 2019). Overexpression of EGFR not only increased the synthesis of Wnt3a, Fzd, and β-catenin, but also deactivated the phosphorylation of multiple Wnt components (Kim N. Y. et al., 2022). Although EGFR inhibitors have not been shown to significantly upregulate Wnt signaling in preclinical investigations, Wnt/β-catenin signaling promotes resistance to EGFR inhibitors in EGFR-mutated LC cells. Previous studies have shown that the canonical Wnt pathway contributes to the maintenance of NSCLC cells during EGFR inhibition and that the efficacy of EGFR inhibitors can be significantly improved by inhibiting various components of the canonical Wnt pathway. Importantly, inhibiting multiple elements of the canonical Wnt pathway can dramatically increase the *in vitro* and *in vivo* effectiveness of EGFR inhibitors (Casás-Selves et al., 2012; Scarborough et al., 2017; Yan et al., 2022). Furthermore, pharmacological reduction of β-catenin nuclear localization inhibited resistance to osimertinib (a third-generation EGFR-TKI) in PC-9 cells resistant to gefitinib (a first-generation EGFR-TKI), and IMU1003, an inhibitor of nuclear localization of β-catenin, significantly reduced the emergence of osimertinib-resistant colonies (Katagiri et al., 2023). This implies that inhibition of nuclear β-catenin function may overcome transgenerational EGFR-TKI resistance.

Aberrant Wnt signaling can inhibit the recruitment of inflammatory anti-tumor T cells and may undermine cancer immunosurveillance, thereby negatively controlling innate immunity, fostering immunoevasion, and boosting resistance to a variety of immunotherapeutics, including immune checkpoint inhibitors (ICIs) (Galluzzi et al., 2019; Hargadon, 2023). Wnt3a overexpression significantly increases the transcriptional activity of the programmed death ligand 1 (PD-L1) promoter reporter (Ma et al., 2022). Molecular screening performed at the time of progression after ICIs treatment in patients with advanced NSCLC revealed molecular alterations in the genes involved in the Wnt pathway, with significantly higher activation of the Wnt pathway in NSCLC during ICIs treatment than before, suggesting a role for this pathway in secondary ICIs resistance (Giroux Leprieur et al., 2020). Inhibition of Wnt signaling, dominated by pharmacological Wnt ligand inhibition, promotes the development of the tumor microenvironment, making it more receptive to checkpoint blocking in patients with LC (DeVito et al., 2021).

## 4 Intervention of natural products on the canonical Wnt pathway in LC

Targeted Wnt therapy has become an important research direction in the development of cancer drugs; however, it also faces problems such as insufficient targeting and large toxic side effects. Therefore, the search for safer and broader drug sources has become the focus of Wnt-targeting research. Natural products have become valuable effective drug sources owing to their wide range of sources, high bioavailability, good safety, and low cost (Sheremet et al., 2017). In the following section, natural products are classified according to their type to discuss their anti-tumor effects via regulation of the Wnt pathway in LC (Table 2). The natural products we discuss in the article are pure compounds extracted from plants except for the Extracts and Chinese Medicine Formulas section.

**TABLE 2:** The intervention for lung cancer Wnt using natural compounds and others.

Group	Name	Mode of action	Effect	Cell type/Modle	References
Flavonoids	EGCG	↓ the expression of p-GSK-3β(Ser9), β-catenin and its downstream target gene c-Myc, ↑GSK-3 expression	↓tumor globular formation. ↓protein and mRNA levels of CSCs markers. ↓proliferation and ↑apoptosis	A549, H1299 cells	Zhu et al. (2017a)
		↑WIF-1 promoter and recovery of WIF-1 expression. ↓β-catenin level and Tcf/Lef reporter activity	potential therapeutic use of reversing the methylation of the WIF-1 promoter	H460, A549 cells	Gao et al. (2009)
	Garcinol	↓phosphorylation of LRP6. ↓β-catenin, Dvl2, Axin2 and Cyclin D1 expression	↓colony formation capacity. ↑cytotoxic death and apoptosis. ↓tumor initiation and growth	H441, A549 cells and H441 CSC mouse xenograft model	Huang et al. (2018)
	Fisetin	↓the expression of various signaling proteins (β-catenin, NF-κB, EGFR, STAT-3) acting on the upstream of EMT	↓EMT program. ↓the migration, invasion potential and stem cell phenotype of LC cells	A549 and H1299 cells	Tabasum and Singh (2019)
	Nobiletin	↓miR-15-5p expression. ↓Wnt/β-catenin	↓stemness and the migration ability. ↑cell apoptosis	A549 and H460 cells	Han et al. (2021)
	TF	↓COX-2, Wnt and β-catenin mRNA expression	↓proliferate and ↑apoptosis of LC cells. ↓tumor growth of mice. ↑survival time of mice	A549 cells, BALB/c nude mouse	Han et al. (2020)
Polyphenols	Curcumin	↓Wnt/β-catenin pathway upregulation mediated by oxidative stress	↓LC cell proliferation	A549	Wang et al. (2018b)
		↓p-GSK3β(Ser9), ↑GSK3β. ↓β-catenin and its downstream c-Myc and Cyclin D1	↓lung CSC proliferation and ↑apoptosis	A549 and H1299 cells	Zhu et al. (2017b)
		↓MTAl-Wnt/β-catenin signal path and ↓downstream targets such as Cyclin D1, and MMP-7	↓proliferation and invasion of NSCLC cells, and ↑G0/G1 phase arrest	95D and A549 cells	Lu et al. (2014)
	RES	↓p-GSK3β(Ser9) β-catenin and c-Myc expression	↓tumor sphere size, expression of CSC specific markers, and activation of Wnt/β-catenin	A549, NCI-H460, NCI-H226 and NCI-H1299 cells, BALB/c nude mice	Xie et al. (2023)
		↑snail stability to reduce Axin2 levels, ↑the binding of β-catenin/TCF to PD-L1 promoter, and of ↑PD-L1 expression	suggesting the role in tumor immune escape	A549 and H1299 cells	Yang et al. (2021a)
	Maclurin	antioxidant activity and ↓Src/FAK-ERK-β-catenin pathway. ↓MMP-2 and MMP-9	↓the migration and invasion of LC cells	A549	Ku et al. (2015)
	Eugenol	regulated the phosphorylation pattern of β-catenin and ↓its nuclear translocation	↓CSC complexity. ↓development of LC and ↑the life span of mice	A549, female Swiss Albino mice	Choudhury et al. (2021)
Alkaloids	Sanguinarine	↓the expressions of Wnt1 and Wnt7b in M2 macrophages	↓tumor growth and angiogenesis in mice	Lewis LC cells, HUVEC, C57BL/6 mice	Cui et al. (2022)
		↓snail and wnt target gene cyclin-D1	↓proliferation, invasion and metastasis of CSC	A549 and H1299 cell lines, nude mice	Yang et al. (2016a)
	Chelerythrine	↓β-catenin cell expression and the localization of β-catenin in the nucleus	↓colony formation and spheroid growth. ↓ability to migrate and invade	NCI-H1703, SK-LU-1, HLCSCs	Heng and Cheah (2020)
	Nuciferine	↑accumulation and stabilization of Axin, ↑degradation of β-catenin and ↓the expression of target genes	↓cell proliferation and apoptosis-resistance. ↓LC growth	A549 and H1299, BALB/c athymic nude (nu/nu) mice	Liu et al. (2015)
	β-asarone	↑GSK-3β expression. ↓protein expression of p-GSK-3β, DVL 2, β-catenin and Cyclin D1. ↓transcriptional activity of β-catenin/TCF	↓tumor cell viability, ↓migration/invasion/adhesion. ↑mitochondria-related apoptosis	A549, NCI-H1299, NCI-H1650 and HCC827 cells	Wang et al. (2018c)
	Lycorine	↑GSK-3β expression. ↓β-catenin and EMT	↓growth and metastasis of lung tumors	A549, H460 cells and A549/Luc mice	Sun et al. (2018)
	Daurisoline	↓the stability of β-catenin and ↑the degradation of β-catenin	↑G1 phase cell cycle arrest. ↓the invasion and metastasis potential of tumor cells and tumor growth in mice	A549, Hop62 and H1299 cells. Female BALB/c nude mice	Huang et al. (2020)
	Matrine	↑Let-7b level. ↓CCND1/Wnt signaling pathway and ↓EMT	↓characteristic labeling and renewal of CSCs and ↑the sensitivity 5-FU. ↓the growth and migration	NCI-A549 and NCI-H460 cells	Li et al. (2020b)
	Ligustrazine	↑GSK-3β expression and ↓the activation of Wnt/β-catenin pathway in tumor tissues	↓tumor formation and development in mice	H1650, A549, H1299 and PC-9 cells	Dong et al. (2020)
				Male BALB/c nude mice	
	Fascaplysin	↓expression levels of β-catenin, AXIN2, GSK-3β and C-myc. EMT phenotype was reversed	↓migration of A549 cells	A549 cells	Luo and Xu (2022)
	(1′S, 6R)-nitidumalkaloid B	↓β-catenin, Axin2, and p-GSK-3β↑p-β-catenin expression	↓tumor cell proliferation, ↑arrest cell cycle and apoptosis	A549 cells	Qin et al. (2023)
Terpenoids	TP	↑methylation of histone H3. ↓expression of various wnt inhibitors (including WIF1, FRZB, SFRP1, ENY2, and DKK1)		A549 and H460 cells. Fen1 E160D transgenic mouse	Nardi et al. (2018)
		the expression level of WIF-1 is increased by demethylation of WIF-1 promoter	↓cell proliferation, invasion, migration and anti-apoptosis	A549 and H460 cells	Mao et al. (2018)
		↑phosphorylation of β-catenin and ↓expression of p-GSK3β(ser9)	↓growth and EMT/metastasis of NSCLC cells	NCI-H1299 and NCI-H460 cells	Deng et al. (2021)
		↓the activity of p70S6K. ↑activated GSK-3 to promote β-catenin degradation	↓the EMT of paclitaxel-resistant LUAD cells	A549 and A549/TaxR cells, (BALB)/c nude mice	Tian et al. (2021)
	CuB	↓Wnt3/Wnt3a expression. ↓GSK-3 Inactivated phosphorylation. ↓β-catenin nuclear localization and TCF-1 interaction	↓cell stemness and metastasis. ↓tumor angiogenesis	A549, H1299 and H23 cells. NNK-induced A/J mouse	Shukla et al. (2016)
	Curcumol	↓LRP5/6 phosphorylation, ↑expression of Axin, APC, GSK-3β and p-β-catenin	↓cell proliferation and survival, motility and migration	A549 and H460 cells. BALB/c nude mice	Li et al. (2021)
	FDN	↓Wnt3 and β-catenin expression levels	↑apoptosis and cell cycle arres	A549 cells	Al Saqr et al. (2022)
	EPBS	↓expression of EGFR, Wnt3a and Fzd-1 which triggered the activation of GSK-3β and induced the degradation of β-catenin	regulated the activity of Wnt/β-catenin pathway proteins in cancer cells with EGFR overexpression and EGFR depletion	A549 and PC-9 cells	Kim et al. (2022a)
	Artemisinin	↓Wnt5a/b, LRP6 Dvl2 and β-catenin expression. ↑NKD2 and Axin2 expression	↑G1 phase cell cycle arrest. ↓tumor proliferation and metastasis	A549 and H1299 cells, Female Balb/c-nude mice	Tong et al. (2016)
	PPD25	↓β-catenin, c-myc, cyclin D1, TCF-4. ↓β-catenin/TCF transcriptional activity	↓proliferation, ↑apoptosis	A549 cells	Bi et al. (2009)
Isothiocyanate	BI and PI	↓β-catenin localization in the nucleus	BI more effectively inhibited long-term colony formation. PI more effectively inhibited the growth of tumor	SK-LU-1, HLCSC	Heng and Cheah (2021)
				NCI-H1703 cells line	
Bufadienolides	19-HB	↓β-catenin c-Myc and CCND1	↓tumor cell viability and ↑apoptosis	NCI-H1299, NCI-H838, A549 cell lines, xenograft mouse model	Yu et al. (2021)
Plant steroid hormone	Epibrassinolide	↓the level of β-catenin. ↓the mRNA levels for a number of genes ( Axin-2, c-Jun, c-Myc, MMP7, MMP9, Nanog	↑cell apoptosis and ↓drug resistance of tumor	VPA17 and H69 SCLC cells	Sadava and Kane (2017)
		Survivin, *etc.*)			
Fungal metabolite	Patulin	↑inhibitors of Wnt pathway, Dkk and WIF-1, ↓Cyclin D1	antiproliferative, proapoptotic, and antimigration effects	A549 cells	Monteillier et al. (2018)
Phenanthrene	Dehydroeffusol	↓expression of β-catenin and its downstream related proteins	↓hypoxic-induced migration and invasion of NSCLC cells	A549 cells	Wei et al. (2020)
Aldehyd	Cinnamaldehyde	↑GSK-3β, p-β-catenin expression. ↓β-catenin, TCF-1 and related genes	↓EMT and growth of NSCLC cells. ↓cell movement and metastasis	A549, YTMLC-90 and NCI-H1299 cells. BALB/c/nu/nu nude mice	Wu et al. (2017)
Carotenoid	Fucoxanthin	↓β-catenin and EMT-associated proteins levels	↓TGF-β1 induced cell migration and invasion and EMT	A549 cells	Luan et al. (2022)
saponin	Ophiopogonin B	↑Axin stabilization to inhibit β-catenin	↓EMT in β-catenin overexpressed cells and↓ movement and metastasis	A549, NCI-H1299 and NCI-H460 cells	Zhang et al. (2021)
Polysaccharide	ASPS	↓the levels of wnt3a, p-GSK-3β and Cyclin D1. ↓the expression of EMT-related markers	↓cell proliferation and metastasis	NCI-H520 cells	Sun et al. (2019)
Derivatives	FV429	↑protein expression of GSK-3β and AXIN. ↓β-catenin nuclear localization	↑G2/M phase arrest induced by paclitaxel	A549 and NCI–H460 cells	Guo et al. (2021)
	MRx102	↑WIF-1 by promoter hypomethylation	had similar effects to triptolide without unwanted toxicity	A549 and H460 cells, NSG Mouse Model	Reno et al. (2016)
	xl, 1c and 8b	↓levels of β-catenin and its downstream targets Cyclin D1, CDK4 and c-myc	↓LC cells and tumor growth	A549 and H460 cells, tumor-bearing mice model	Bi et al. (2014)
	DUD	↑Axin expression and p-β-catenin proteolysis	reversed EMT and inhibited migration	A549 cells	Dai et al. (2020)
	12K	↓LRP5/6 phosphorylation and ↓β-catenin expression and nuclear translocation	↓cell growth and metastasis	A549 cells	Dai et al. (2017)
Extracts	GBEE	↓expression of Wnt3a and β-catenin. ↓levels of VEGF and VEGFR2	↓tumor growth and metastasis. ↓MVD in LC grafts	Lewis LC cells, C57BL/6 nude mice	Han et al. (2016)
	AvL-EtOH	↓Wnt3a, β-catenin and downstream genes	↑Caspase-mediated apoptosis and ↓proliferation	A549 cells	Tiwari et al. (2023)
	Atranorin	↓nuclear import of β-catenin and downstream genes	↓invasion and inhibit tumor growth↓tumorigenesis	A549, H460, H1650 and LLC cells, C57BL/6 mice	Zhou et al. (2017)
Chinese medicinal formulae	Miao	↓the expression of c-myc, AXIN and β-catenin	Miao and cisplatin can synergistically regulate LC cell proliferation and apoptosis	NCI-H446 cells, C57BL/6 nude mice	Li et al. (2020a)
	QYSL	↓Wnt1、Wnt2、Wnt5a、GSK3β、p-GSK-3β、β-catenin and Dvl-1	synergistic anti-tumor effect with cisplatin	Lewis lung carcinoma cell xenografts model in nude mice	Tong et al. (2018)
	FYLM	↓expression of p-EGFR, PRC1 and Wnt pathway-related proteins such as β-catenin, c-Myc and c-Jun	↑EGFR-mutated NSCLC cells sensitive to osimertinib	EGFR triple-mutant Lewis LC cells and xenograft mouse model	Shi et al. (2023)
	FYN	↓GSK-3β and β-catenin	↓proliferation and migration of Osimertinib-resistant cell lines	HCC827OR, PC9OR cells, xenografts model in nude mice	Sang et al. (2022)

### 4.1 Flavonoids

Flavonoids, which are frequently found in human diets and have biological effects, including antioxidant, antibacterial, and anti-inflammatory properties, can lower the risk of disease and are thought to be natural tumor-preventive agents. The biological activity of flavonoids relies on the structural substitution of the C6-C3-C6 loop (Shen et al., 2022). The following is a review of relevant literature on Epigallocatechin gallate (EGCG), garcinol, fisetin, nobiletin, and total flavonoids (TF) involved:

EGCG, the main bioactive ingredient in green tea, is linked to a number of bioactivities, including, anti-cancer, antioxidant, anti-inflammatory and antibacterial activities (Zhang S. et al., 2023). The inactive form of GSK-3β, which is phosphorylated at Ser9 and inhibited by EGCG, became significantly more abundant in the presence of EGCG, while β-catenin and Myc decreased. EGCG increased apoptosis, prevented lung CSCs from proliferating, and decreased the expression of lung CSCs markers at both the protein and mRNA levels (Zhu J. et al., 2017). Lithium chloride (LiCl) is a typical Wnt activator. By causing its phosphorylation at ser9, LiCl therapy inactivated GSK-3β to enhance β-catenin activation which overrode the inhibitory effects of EGCG on the Wnt pathway, tumor sphere formation, and lung CSC markers. LiCl treatment decreased EGCG’s ability of EGCG to inhibit the growth of lung CSCs and induce apoptosis (Zhu J. et al., 2017). In addition, aberrant promoter methylation of WIF-1 is the basic mechanism of epigenetic silencing in human cancers. Previous studies have also shown that EGCG treatment can regain WIF-1 expression by promoter demethylation of WIF-1 in NSCLC cell lines (Mazieres et al., 2004). Additionally, cytosolic β-catenin protein levels are lowered by EGCG, and TCF/LEF reporter activity is suppressed (Gao et al., 2009).

Garcinol, a polyisoprenylated benzophenone derived from *Garcinia indica* Choisy (kokum) fruiting bodies and having anti-inflammatory, antioxidant, acetyltransferase inhibitory, and anti-cancer effects, alters lung CSCs’ activity and the aggression that goes along with them. Garcinol interferes with the phosphorylation of LRP6, a shared Wnt and Stat3 receptor, and blocks the activation of the Wnt/β-catenin/Stat3 axis. In NSCLC-produced spheres, garcinol reduced the expression of Dvl2, Axin2, β-catenin, and CCND1, indicating that garcinol can control the Wnt/β-catenin signaling pathway. Garcinol therapy dramatically slowed the formation of tumors in a mouse model of H441 CSC, confirming the above findings *in vivo*. Garcinol was found to modulate the lung CSCs phenotype by modulating Wnt/β-catenin signaling and Stat3 inactivation, thus suggesting that garcinol might be a potential new anti-lung CSC medicinal substance (Huang et al., 2018).

Fisetin (3,3′,4′,7-tetrahydroxyflavone) is a natural bioactive flavonoid present in a variety of fruits and vegetables, displaying a range of pharmacological properties, such as anti-invasive and anti-cancer actions (Sundarraj et al., 2018). Fisetin controls the expression of EMT markers and lowered the expression of several signaling proteins that act upstream of EMT such as β-catenin, EGFR, and Stat3. It also decreases the expression of the invasion markers MMP-2, vimentin, and N-cadherin, thereby limiting the potential of the tumor to metastasize. Both the stem cell-like phenotype of H1299 cells and the expression of the lung CSC markers CD44 and CD133 were downregulated following fisetin treatment. Fisetin also inhibited LC cell proliferation by inducing G1-phase cell cycle arrest instead of cell death (Tabasum and Singh, 2019).

The polymethoxylated flavonoid nobiletin (3′,4′,5,6,7,8-hexamethoxyflavone) is specifically accumulated in citrus fruit peel, with many anti-cancer activities. Nobiletin was found to enhance regulators of Wnt/β-catenin signaling, such as Axin2 and WIF-1, which are related to the inhibition of miR-15-5p expression. Nobiletin significantly reduced the ability of NSCLC cells to form spheres and the expression of proteins linked to cancer stemness, namely, CD133 and ALDH1 (Han et al., 2021). Study also discovered that nobiletin prevented A549 and H460 cells from migrating, demonstrating a strong anti-metastatic effect that may be related to the suppression of cell stemness and EMT induced by the inactivation of the Wnt pathway (Han et al., 2021).

TF isolated from Fructus viticis significantly reduced the mRNA expression levels of cyclooxygenase 2 (COX-2), Wnt, and β-catenin in A549 cells. Further research found that TF promoted the apoptosis of A549 cells by inhibiting the COX-2 mediated Wnt/β-catenin signaling pathway, increasing multiple pro-apoptotic genes expression such as apoptotic peptidase activating factor 1, and lowering expression of anti-apoptotic genes BCL2L2 and BCL2. The ability of A549 cells to proliferate was decreased by TF in a dose-dependent manner; however, this effect was reversed by activating Wnt signaling via COX-2. Additionally, animal studies revealed that TF therapy greatly slowed the growth of mouse tumors, with a tumor inhibition rate of 64.07%, and increased the survival time of the animals (Han et al., 2020).

### 4.2 Polyphenols

Phenols in large numbers and diverse forms provide a variety of potential benefits to human health. Polyphenols have multiple conjugated double bonds and show strong antioxidant capacities. Therefore, when electrons at certain sites are removed by free radicals, the molecular structure is stabilized by conjugation, thereby protecting the cell (Sharoni et al., 2012). This review discusses curcumin, resveratrol, and other compounds (maclurin and eugenol).

#### 4.2.1 Curcumin

Oxidative stress is one of the most common causes of cellular damage, mainly due to the accumulation of reactive oxygen species (ROS) and other free radicals causing damage to antioxidant enzymes such as superoxide dismutase (SOD) (Sies et al., 2017). Previous studies have demonstrated that the Wnt/β-catenin pathway can be activated by ROS-mediated induction, and that the crosstalk between ROS and the Wnt pathway plays an important role in LC adeno-to-squamous transdifferentiation (Fang et al., 2023). The primary component of turmeric (*Curcuma longa* L.), a commonly used traditional Chinese herbal remedy, is curcumin (diferuloylmethane), which has numerous pharmacological effects, including anti-tumor, anti-inflammatory, antioxidative, and neuroprotective properties (Deng et al., 2022). Curcumin reduced ROS levels, increased SOD and γ-glutamyl cysteine synthetase levels, and inhibited A549 cell growth. Curcumin also significantly reduced the expression of nuclear β-catenin and phosphorylated GSK-3β (p-GSK3-β) in their respective proteins. Curcumin treatment drastically inhibited CCND1 and Myc expression. Pretreatment with an ROS scavenger (NAC) significantly reversed this effect. It is clear that oxidative stress plays a role in curcumin-induced suppression of the Wnt/β-catenin pathway by curcumin (Wang J. Y. et al., 2018).

Another study discovered that curcumin effectively reduces lung CSC activity by suppressing the Wnt/β-catenin and Sonic Hedgehog pathways to diminish tumor sphere formation, downregulate CSC marker expression, inhibit proliferation, and induce apoptosis. LiCl-triggered Wnt/β-catenin activation reversed the anti-tumor effects of curcumin (Zhu J. Y. et al., 2017). Curcumin can reduce the transcription and translation of metastasis-associated protein 1 (MTA1) in LC cells and increase the number of cells in the G0/G1 phase. In addition, MTA1 knockout affected the protein levels of β-catenin, MMP7, and CCND1, which was similar to the effect of curcumin treatment in NSCLC. These results suggest that curcumin inhibits the proliferation and metastasis of 95D and A549 cells by reducing their downstream targets via inactivation of the MTAl-Wnt/β-catenin pathway (Lu et al., 2014). Furthermore, the inhibitory impact of curcumin on NSCLC cells may also be attributed to overexpression of miR-192-5p, which targetd c-Myc and deactivated the Wnt/β-catenin signaling pathway (Pan et al., 2020).

#### 4.2.2 Resveratrol

The bioactive phytochemical resveratrol (RES), which is obtained from a variety of plants, has tumor-suppressing effects on LC. RES successfully reduced IL-6 expression and the levels of the CSC-specific markers CD133, ALDH1A1, and Nanog in NSCLC cell lines. Moreover, RES dose-dependently decreased the expression of β-catenin, p-GSK-3β (Ser9), and Myc and inhibited Wnt/β-catenin signaling *in vivo*, which increased the lifespan of mice and prevented the growth of LC. The addition of LiCl partially counteracts the inhibitory effect of RES on lung CSCs and reverses the inhibition of Wnt/β-catenin signaling by RES (Xie et al., 2023). Interestingly, the Wnt pathway was found to be essential for mediating RES-induced upregulation of PD-L1. In H1299 cells, RES reduced Axin2 levels by increasing Snail stability, leading to breakdown of the destruction complex and enhancing TCF/β-catenin adherence to the PD-L1 promoter. RES dose-dependently increases PD-L1 expression in NSCLC cells at pharmacologically accessible concentrations (<30 μM), and RES treatment strongly inhibits T cell function in a co-culture model (Yang M. et al., 2021). These findings imply that RES may contribute to tumor immune evasion.

These contradictory results may be attributed to the different concentrations of RES. PD-L1 expression was induced after exposure to low doses of resveratrol in H1299 cells. However, at higher doses (>40 μM), RES treatment resulted in a gradual reduction of PD-L1, suggesting that RES can modulate PD-L1 expression in a dose-dependent manner (Yang M. et al., 2021). More importantly, there are two structures of natural RES, cis (3,5,4′-Trihydroxystilbene) and trans (trans-3, 5,4′-trihydroxystilbene), which have significant metabolic differences, and may have different or even opposite effects on cells. Further investigation is required to fully understand the possible advantages and hazards of using RES as an adjuvant cancer therapy (Sundarraj et al., 2018; Jarosova et al., 2020; Jhanji et al., 2021).

#### 4.2.3 Other polyphenols

*Morus alba* L. and *Garcinia mangostana* L. are sources of the natural compound maclurin ((3,4-dihydroxyphenyl)-(2,4,6-trihydroxyphenyl)methanone), a member of the benzophenone family (Moon et al., 2022). The antioxidant activity of maclurin significantly reduced intracellular ROS levels, leading to the inhibition of Src/FAK and ERK in A549 cells. Subsequently, attenuated GSK-3β and reduced accumulation of nuclear β-catenin were observed after maclurin treatment, which further caused downregulation of transcriptional expression and activation of metastasis-related MMP-2 and MMP-9, leading to the inhibition of migration and invasion of human NCLC cells (Ku et al., 2015).

Eugenol (4-allyl (-2-methoxyphenol)) is abundant in the essential oils of widely used Asian species, such as cloves. Eugenol, a naturally occurring β-catenin inhibitor, is an extremely important chemopreventive agent for LC. Eugenol can sequester β-catenin in the cytoplasm and regulate the phosphorylated form of β-catenin to promote its degradation, thus helping the cellular environment to resemble the normal state. Meticulous molecular analysis clearly revealed GSK-3β-mediated phosphorylation of β-catenin after eugenol treatment, leading to degradation of cytoplasmic β-catenin, which significantly limited its transfer to the nucleus. Eugenol activity reduced the expression of CSC markers regulated by β-catenin (CD44, Oct4, EpCAM, and Notch1). Eugenol successfully slowed the activity of the small-molecule CK1 inhibitor D4476, perpetuating the mandatory phosphorylation of β-catenin by CK1α, thereby pushing it toward degradation and minimizing CSC expression. Eugenol treatment showed tumor-preventing effects and significantly prolonged the lifespan of mice with N-nitrosodiethylamine-induced LC (Choudhury et al., 2021).

### 4.3 Alkaloids

Natural alkaloids are regarded as valuable sources of anti-cancer medicines because they not only exhibit potent anti-tumor effects when taken alone but also when combined with other anti-tumor agents (Luo et al., 2021). Consequently, alkaloids have attracted considerable interest for tumor treatment.

Sanguinarine, a benzophenanthrodine alkaloid, exerts anti-tumor effects through multiple targets and pathways, such as the CSC and TAM phenotypes. A tappable driver of the immunosuppressive M2-like TAM phenotype is the Wnt signaling pathway, which can be regulated by sanguinarine. In M2 macrophages, sanguinarine reduced the protein expression of the nuclear transcription factor β-catenin as well as the mRNA expression of Wnt1 and Wnt7b. By preventing Wnt signaling in TAMs, sanguinarine controlled M2 macrophage polarization and inhibited angiogenesis, showing potential application value in immunotherapy and anti-angiogenesis therapy for LC (Cui et al., 2022). In addition, sanguinarine inhibited the proliferation, invasion, and metastasis of lung CSC mainly through direct downregulation of Wnt/β-catenin self-renewal pathway and indirect interruption of EMT. Sanguinarine, which can be used to target lung CSC, decreased the expression of β-catenin and downstream CCND1 (Yang et al., 2016a). Moreover, a possible anti-cancer target of sanguinarine *in vitro* is the interaction between β-catenin and LEF1, based on a high-throughput sequencing assay (Chen et al., 2021).

The type III benzophenanthridine alkaloid Fisetin was discovered in medicinal plants including *Chelidonium majus* L. and *Macleaya cordata* (Willd.) R. Br (Liu et al., 2006). The major histology of lung CSCs is sensitive to the growth inhibitor chelerythrine chloride, which inhibits the growth of LC cell lines derived from different proximal/distal origins. By downregulating β-catenin, it suppressed the function of CSCs and triggered apoptosis in a variety of LC cells with diverse histological backgrounds. Chelerythrine chloride caused not only a reduction in average β-catenin cellular expression, but also a reduction in nuclear β-catenin localization in several lung tissues, regardless of tissue origin, and different concentrations could trigger the specific targeting required in different cell lines. This broad applicability of chelidonine chloride as a β-catenin inhibitor would favor its development as an anti-CSC therapy for LC (Heng and Cheah, 2020).

Nuciferine (NF), an alkaloid originally isolated from lotus (*Nelumbo nucifera* Gaertn.) leaves, has therapeutic effects such as anti-obesity, antioxidant, and anti-inflammatory properties (Kim S. M. et al., 2022). Axin protein levels significantly increased in NF-treated cells, but not APC or GSK-3 expression levels, while Axin, APC, or GSK-3 mRNA levels were unaffected. The effect of NF was sufficient to induce the accumulation and stabilization of Axin, which accelerated β-catenin degradation and inhibited Wnt/β-catenin signaling in NSCLC cells. NF effectively inhibited the proliferation of nicotine-pretreated NSCLC cells and exerted chemotherapeutic effects on tumor xenograft growth *in vivo*. NF effectively inhibited the proliferation of NSCLC cells pretreated with nicotine *in vitro* and exerted chemotherapeutic effects on tumor xenograft growth *in vivo* (Liu et al., 2015).

β-asarone (1-propenyl-2,4,5-methoxybenzol), the main bioactive component of *Acorus calamus* L., possesses diverse pharmacological activities, including anti-cancer, antidepressant, antiepileptic, antihyperlipidemic, antithrombotic, and radioprotective activities (Chellian et al., 2017). β-asarone induced a dose-dependent decrease in lung carcinoma cell viability, cell migration, invasion and adhesion, and induced tumor mitochondria-associated apoptosis through an endogenous pathway, which significantly increased the activities of caspase-3 and caspase-9. β-asarone increased the expression of GSK-3β while downregulating the expression of Dvl2, p-GSK-3β (Ser 9), and β-catenin. β-asarone treatment also impaired the transcriptional activity of β-catenin/TCF and the expression of its downstream target genes, including MMP-7, Myc, and CCND1. Restoring Wnt/β-catenin signaling pathway activation by Wnt3a can overcome the anti-LC effect induced by β-Asarone (Wang T. L. et al., 2018).

The natural isoquinoline alkaloid lycorine, which is extracted from the herb *Lycoris radiata* (L’Her.), has many anti-cancer properties. After treatment with lycorine, the expression of GSK-3β was increased, the cellular level of β-catenin was inhibited, and the growth and metastasis of LC were significantly hindered. This is closely related to the inhibition of Wnt/β-catenin signal transduction by lycorine and the reversal of EMT in LC cells (Sun et al., 2018).

Daurisoline, an alkaloid constituent of *Menispermum Dauricum* DC., has excellent anti-inflammatory properties (Zhou et al., 2022). Daurisoline may directly target HSP90, preventing its interaction with β-catenin and accelerating β-catenin’s ubiquitin-mediated proteasomal destruction. Daurisoline decreased the expression of the downstream genes Myc and CCND1, resulting in cell cycle arrest at the G1 phase. It also influenced the expression of mesenchymal markers, such as N-cadherin and vimentin, which impair the invasive and metastatic potential of LC cells. Daurisoline inhibits the tumorigenicity of LC both *in vitro* and *in vivo* (Huang et al., 2020).

Matrine is the main bioactive component of *Sophora flavescentis* Ait. and has been widely used clinically in China, either alone or in combination with chemotherapeutic drugs or radiotherapy (Li et al., 2016b). Matrine regulates CSCs renewal in NSCLC in a CCND1/Wnt signaling-dependent manner. By targeting the Wnt/CCND1 pathway to prevent 5-fluorouracil-induced CCND1 accumulation in tumor tissues, matrine sensitized NSCLC cells to the effects of 5-fluorouracil *in vivo* and markedly boosted apoptosis. In addition, matrine alleviated EMT by increasing the expression of E-cadherin and lowering the level of N-cadherin, and CCND1 knockdown in NSCLC cells reduced EMT. Matrine regulates CCND1/Wnt-mediated EMT and decreases the mobility of NSCLC cells (Li X. et al., 2020).

The alkaloid ligustrazine is an active component of the Chinese herbal remedy *Ligusticum chuanxiong* Hort. By increasing the level of GSK-3β, ligustrazine considerably reduced the weight and volume of mouse tumors and significantly blocked the activation of the Wnt/β-catenin pathway in LC tissue. This resulted in the inhibition of tumor growth in mice (Dong et al., 2020).

Natural substances derived from sponges, such as fascaplysin, exhibit promising anti-cancer properties. Fascaplysin blocks the cell cycle of A549 cells by regulating cycle-related proteins such as cyclin D1. Fascaplysin treatment reduced the expression levels of Axin2, GSK-3β, β-catenin, and Myc proteins, which inhibited LC cell migration by hindering Wnt signal transduction and reversing the EMT phenotyp (Luo and Xu, 2022).

(1′S, 6*R*)-nitidumalkaloid B is a novel alkaloid identified from *Zanthoxylum nitidum* (Roxb.) DC. and its antiproliferative activity is partly related to G2/M cell cycle arrest, induction of apoptosis and inhibition of the Wnt/β-catenin signaling pathway. It effectively suppressed the expression of β-catenin, Axin2, and p-GSK-3β but increased p-β-catenin expression in a concentration-dependent manner. Constitutive relationship analysis suggested that the C-6 substituent of benzophenanthridine alkaloids had a direct effect on its antiproliferative activity (Qin et al., 2023).

### 4.4 Terpenoids

Terpenoids, also known as isoprenoids, are the most diverse compounds in nature, accounting for approximately one-third of all natural products, and are widely used in the field of medicine. For example, paclitaxel is a widely used chemotherapeutic drug in clinical practic (Christianson, 2017). Recently, some terpenoids have shown beneficial effects on the Wnt pathway in LC.

#### 4.4.1 Triptolide

Triptolide (TP), an important active compound in *Tripterygium wilfordii* Hook. f., is a natural diterpene epoxy compound with strong anti-cancer activity that has been widely studied. TP-induced epigenetic changes in histone H3 methylation and nuclear translocation of β-catenin effectively inhibited Wnt signaling in multiple LC models, thereby inhibiting the proliferation, metastasis, and drug resistance of LC cells. The expression of several Wnt inhibitors, such as WIF-1 and DKK1, significantly increased following treatment with TP (Nardi et al., 2018). After treatment with TP, the demethylation of WIF-1 promoter increased the mRNA and protein levels of WIF-1, which prevented the excessive activation of Wnt pathway and affected the downstream Axin2 and β-catenin expression (Mao et al., 2018). Triptolide treatment promoted β-catenin phosphorylation and decreased p-GSK-3β(ser9) expression, inhibited EMT in the xenograft model and reduced lung metastasis in the tail vein injection model. β-catenin overexpression antagonized the triptolide-mediated inhibition of EMT marker expression and counteracted the triptolide-mediated inhibition of the migration and invasion of LC cells (Deng et al., 2021). TP promoted the degradation of β-catenin by blocking the activity of the protein kinase p70S6K, which was found to inactivate phosphorylated GSK-3 at serine 9/21. By repressing the Wnt/β-catenin pathway, TP reversed EMT in paclitaxel-resistant lung adenocarcinoma cells, offering a prospective use for TP and paclitaxel combination therapy (Tian et al., 2021).

#### 4.4.2 Other terpenoids

Cucurbitacin B (CuB) is a natural plant triterpenoid that inhibits endothelial cell migration, invasion, and angiogenesis in NSCLC. CuB treatment inhibited the expression of Wnt3/Wnt3a, inactivating GSK-3 phosphorylation, and restricted β-catenin nuclear localization to influnce interaction with TCF-1 in NSCLC cells. Downregulation of the Wnt/β-catenin signaling axis conferred the ability of CuB to resist NSCLC stemness and metastasis. Additionally, CuB showed strong antiangiogenic properties both *in vivo* and *in vitro*. This property may be attained by influencing angiogenic mediators such MMP-2, survivin, and VEGF in Wnt target genes (Shukla et al., 2016).

The sesquiterpene Curcumol, a bioactive substance purported to have anti-cancer effects, was isolated from rhizome of various Curcuma species. Curcumol treatment controlled the motility, migration, and proliferation of LC cells by altering the expression of Wnt signaling molecules. Curcumol inhibited the phosphorylation of LRP5/6, although Fzd8 was not altered, thereby greatly increasing the protein expression of downstream Axin, APC and GSK-3β. Thus, the β-catenin protein expression was dramatically reduced while its degree of phosphorylation was significantly higher (Li et al., 2021).

Sesquiterpenoids isolated from Curcuma species also contain furanedienone, which may be a potential adjuvant therapy for the treatment of LC. In A549 cells, furanodienone administration suppressed Wnt3 and β-catenin in a dose-dependent manner, thereby downregulating the Wnt signaling pathway. This was correlated with increased apoptosis and cell cycle arrest (Al Saqr et al., 2022).

Euphorbiasteroid (EPBS), a tricyclic terpenoid extracted from *Euphorbia lathyrism* L., inhibited the expression of EGFR, Wnt3a, and Fzd-1 in NSCLC cells and triggered the activation of GSK-3β, which in turn induced β-catenin degradation. In EGFR-overexpressing and-depleted LC cells, EPBS regulated the constitutive activation of the Wnt/β-catenin pathway, and the nuclear translocation of β-catenin was considerably decreased upon EPBS administration, suggesting that EPBS may be implemented alongside EGFR-TKIs in drug-resistant LC (Kim N. Y. et al., 2022).

The antimalarial compounds artemisinin, dihydroartemisinin, and artesunate halt lung tumor progression by blocking the Wnt/β-catenin pathway. They suppressed Wnt5-a/b protein levels and efficiently lowered the expression of LRP6 and Dvl2 while increasing Axin2 expression. Ultimately, this results in downregulation of β-catenin and inhibition of LC proliferation and metastasis (Tong et al., 2016).

20(S)-25-OCH3-PPD (PPD25), a dammarane-type triterpenoid saponin from the leaves of Panax notoginseng (Burk.) F. H., has been reported to have cytotoxic effects in several human cancer cell lines. PPD25 dose-dependently decreased β-catenin expression and inhibited β-catenin/TCF transcriptional activity in A549 cells. It inhibited the expression of Wnt pathway-associated proteins and effectively suppressed proliferation of LC cells, demonstrating the potential as a chemotherapeutic and/or chemopreventive agent for LC (Bi et al., 2009).

### 4.5 Other types

Isothiocyanate members reduce the signature activation event of β-catenin nuclear localization in LC cell lines. Phenethylisothiocyanate was more effective in preventing the growth of a multicellular tumor spheroid model that mimicked micrometastases, whereas Benzyl isothiocyanate was better at preventing the Long-term colony formation dependent on CSC viability in Wnt-dependent squamous cells. Therefore, Phenethylisothiocyanate might work as chemotherapy to remove a large tumor and Benzyl isothiocyanate may be a safer alternative to target undifferentiated CSCs (Heng and Cheah, 2021).

19-Hydroxybufalin (19-HB) is a monomer of bufadienolides from the skin of toads. 19-HB reduced β-catenin as well as downstream target genes. It produced safe anti-tumor effects *in vitro* and *in vivo* by decreasing viability and promoting apoptosis of LC cells, and its anti-tumor effects were mediated by inhibition of the Wnt signaling pathway (Yu et al., 2021).

Epibrassinolide is a steroid hormone in plants that acts via membrane receptors and GSK-3 pathway, resulting in stabilization of transcription factors. It significantly reduced β-catenin-dependent cytokinesis, metastasis, and anti-apoptotic gene expression in both drug-resistant (VPA17) and drug-sensitized (H69) small cell LC (SCLC) cells, but had no effect on normal lung epithelial cells (Sadava and Kane, 2017).

Patulin, a metabolite of *Penicillium vulpinum*, exhibited antiproliferative, proapoptotic, and antimigration effects on human LUAD cells through inhibition of the Wnt pathway. It upregulated the expression of two endogenous inhibitors of Wnt pathway, Dkk and WIF-1, and downregulated the expression of CCND1 (Monteillier et al., 2018).

Other compounds, such as dehydroeffusol (Wei et al., 2020), cinnamaldehyde (Wu et al., 2017), fucoxanthin (Luan et al., 2022), ophiopogonin B (Zhang et al., 2021) and acanthopanax senticosus polysaccharide (ASPS) (Sun et al., 2019), can inhibit the Wnt pathway to weaken the EMT process and inhibit tumor metastasis.

### 4.6 Derivatives

In addition, some derivatives artificially manufactured based on the well-defined structures of pure compounds extracted from plants. Most of them showed better bioavailability and achieved better anti-tumor effects by enhancing efficacy and attenuating toxicity.

FV-429, a derivative of the natural flavonoid wogonin, reprogrammed cancer cell metabolism and reduced fatty acid levels. The combination of FV-429 and paclitaxel not only reduced the levels of Wnt3a, LRP6, and Dvl 2 but also increased the protein expression of GSK-3β and Axin, which blocked the hypoxia-driven nuclear localization of β-catenin. Furthermore, FV-429-regulated fatty acid metabolism enhanced the efficacy of paclitaxel in NSCLC through Wnt-mediated G2/M phase arrest (Guo et al., 2021).

The triptolide derivative MRx102 upregulated WIF-1 in LC cell lines via hypomethylation of its promoter. Interestingly, MRx102 has effects similar to those of triptolide without unwanted toxicity (Reno et al., 2016). Three ginsenoside derivatives, xl, 1c, and 8b, showed good anti-tumor activity against LC, and their anti-growth activity against LC cells partly involved β-catenin-mediated signaling, as these substances reduced the expression of β-catenin and its subsequent targets CCND1, CDK4, and Myc (Bi et al., 2014).

Based on the structure of the natural product taspine, a diphenylurea derivative (DUD) was synthesized using a computer-aided drug design method to reduce the expression and nuclear translocation of β-catenin. DUD significantly upregulated Axin expression and stabilized the β-catenin destruction complex to promote proteasome-mediated protein hydrolysis of phosphorylated β-catenin. DUD reversed EMT process and inhibited LC cell migration via Wnt/β-catenin and PI3K/Akt signaling (Dai et al., 2020). Compound 12k, a derivative of taspine, adversely controlled the Wnt signaling pathway by reducing the nuclear translocation of β-catenin and suppressing the phosphorylation of LRP5/6. Compound 12k downregulated MMP3 and MMP7 expression, which was controlled by β-catenin in A549 cells. Related migration proteins such as VEGF, MMP2, and MMP9 were impaired after 12k treatment (Dai et al., 2017).

### 4.7 Extracts and Chinese Medicine Formulas

Ginkgo biloba exocarp extract (GBEE) contains a large number of components with bioactivities, such as antioxidant, antibacterial and anti-tumor. Ginkgo biloba seed exocarp polysaccharides and ginkgolic acids are be identified as the main components in GBEE contributing to the anti-tumor effect (Wang et al., 2022). GBEE significantly inhibited the expression of Wnt3a and β-catenin in mice and reduced VEGF and VEGFR2 mRNA levels in LC cells. GBEE inhibits Lewis LC angiogenesis by inhibiting the Wnt/β-catenin-VEGF signaling pathway to suppress MVD in transplant tumors which inhibits tumor growth and suppresses tumor lung metastasis in a dose-dependent manner (Han et al., 2016).

The ethanolic extract of *Artemisia vulgaris* L. (AvL-EtOH) decreased the downstream activation of Wnt3a in A549 cells, thereby inhibiting tumor cell proliferation and promoting caspase cascade activation-induced apoptosis. The gas chromatography-mass spectrometry analysis of AvL-EtOH exhibited 48 peaks of various secondary metabolites such as terpenoids, flavonoids, carbohydrates, coumarins, amino acids, steroids and proteins (Tiwari et al., 2023).

Everniastrum vexans is a secondary metabolite extracted from lichen with anti-migration activity to LC cells, and atranorin is identified as an active subcomponent of this extract. Atranorin inhibited LC cell motility and tumorigenic potential in xenograft tumor model by inhibiting the nuclear import of β-catenin and down-regulating downstream genes expression (Zhou et al., 2017).

Anti-cancer formulas, such as Miao-Yi-Ai-Tang (Miao) and Qiyusanlong (QYSL) decoction, significantly enhance the effect of Cisplatin on LC cells. Miao treatment inhibited the expression of Axin, β-catenin, and Myc, restricting the development of LC. Miao not only had similar effects as cisplatin on LC cells, but also induced an improvement in cisplatin resistance. The simultaneous use of Miao and cisplatin synergistically regulated cell proliferation and apoptosis and enhanced the inhibitory effect on LC cells (Li B. et al., 2020). QYSL inhibited Wnt1, Wnt2, Wnt5a, GSK-3β, p-GSK-3β, β-catenin, and Dvl-1 protein levels and decreased downstream expression of related mRNAs. The combination of chemotherapy drug Cisplatin and a high dose of QYSL decoction significantly enhanced the inhibitory effect on LC (Tong et al., 2018).

The Feiyiliu Mixture (FYLM) and Feiyanning Formula (FYN) are experienced Chinese medicinal formulas. FYLM reduced the expression of phosphorylated EGFR and Wnt pathway-related proteins, such as β-catenin and Myc. FYLM treatment delays the resistance of EGFR-mutant cell lines and EGFR-mutant tumor-bearing mice to osimertinib by inhibiting the Wnt/EGFR pathway (Shi et al., 2023). FYN in combination with osimertinib significantly inhibited the proliferation and migration of drug-resistant NSCLC cell lines. It reduced GSK-3β and β-catenin expression and exerted anti-osimertinib resistance by inhibiting the Wnt/β-catenin pathway (Sang et al., 2022).

**FIGURE 2:**
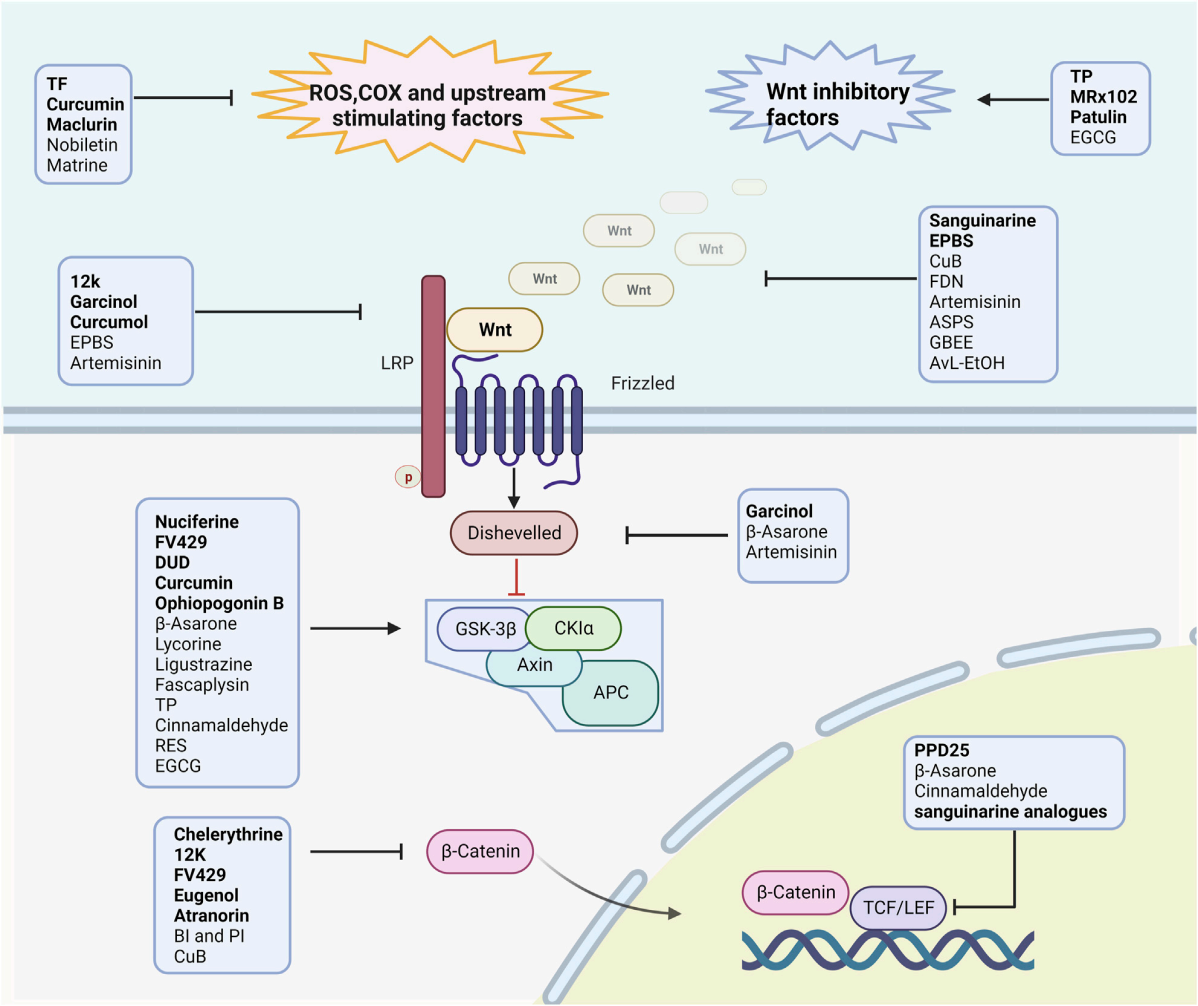
The manner in which the studied product acts on the Wnt pathway in lung cancer. Natural compounds and their derivatives inhibit the Wnt signaling pathway by reducing upstream activators such as SOX and COX-2, inhibiting Wnt ligands, inhibiting receptor phosphorylation such as Fzd, inhibiting Dvl, promoting destruction complex stabilization, and inhibiting β-catenin nuclear localization and TCF/LEF transcription factor activity.

Additionally, almost every element of the Wnt signaling pathway, from the synthesis and secretion of Wnt ligands to the transcription of Wnt target genes, has the potential to become a molecular target for cancer therapy (Sheridan, 2018). Natural products and their derivatives could target various components of the canonical Wnt pathway (Figure 2) and have a high safety profile. Some can enhance efficacy or inhibit drug resistance, showing good potential for combination therapy with existing anti-tumor drugs (Table 3). This may be an important breakthrough point to address the limited therapeutic window, and provide new avenues for LC treatment.

**TABLE 3:** Effectiveness of subjects in combination with existing drugs.

Name	Anti-LC drugs	Effect of combination	References
Fisetin	Erlotinib	enhance the cytotoxic effect of the drug	Tabasum and Singh (2019)
Matrine	5-fluorouracil	matrine increased the sensitivity of lung CSCs to 5-FU and inhibited the accumulation of CCND1 in tumor tissues induced by 5-FU.	Li et al. (2020b)
TP	Paclitaxel	reversing EMT and impairing tumor growth in paclitaxel-resistant LUAD cells	Tian et al. (2021)
EPBS	-	modulation of Wnt pathway protein activity in EGFR overexpressing and EGFR depleted cancer cells with potential for anti-TKI resistance	Kim et al. (2022a)
Epibrassinolide	Etoposide	the combination index showed synergism between epibrassinolide and etoposide and between epibrassinolide and doxorubicin. Reversing resistance to two chemotherapeutics	Sadava and Kane (2017)
	Doxorubicin		
FV-429	Paclitaxel	remodeling of fatty acid metabolism in hypoxia-induced paclitaxel-resistant NSCLC through inactivation of the Wnt pathway and increasing sensitivity to paclitaxel	Guo et al. (2021)
Miao	Cisplatin	simultaneous treatment can significantly enhance the effect of DDP on LC cells. It can synergistically regulate the proliferation and apoptosis of lung cancer cells	Li et al. (2020a)
OYSL	Cisplatin	treatment with QYSL broth alone was milder compared to DDP, however, the combination of QYSL broth and chemotherapy demonstrated enhanced anti-tumor effects compared to treatment administered alone	Tong et al. (2018)
FYLM	Osimertinib	sensitizing EGFR Del19/T790M/C797S mutant NSCLC cells to osimertinib	Shi et al. (2023)
FYN	Osimertinib	synergistic effect against cell proliferation and migration. Inhibit drug resistance	Sang et al. (2022)

## 5 Drug delivery systems

Although natural products and their derivatives have achieved promising anti-tumor effects in basic research, they still face challenges in clinical applications, such as low solubility, limited oral bioavailability, poor metabolism and insufficient organ targeting. Because of these limitations, most the natural products have to apply to patients with a huge dosage, which may increase the risk of inducing side effects (Atanasov et al., 2021). Therefore, it would be crucial to research effective drug delivery systems (DDSs) for these compounds.

Nanotechnology helps reduce chaotic distribution and direct drug containment in cancer cells through the movement of DDSs to a greater extent, increasing local utilization, maintaining a higher bioactivity and improving metabolic stability (Hu et al., 2017; Maqsoudlou et al., 2022). The immature vascular system with large cellular gaps in LC has enhanced permeability and retention of nanosized DDSs. The smaller capillaries in the lung enable passive lung targeting of DDSs larger than 7 µm in size (Park et al., 2019). It has been suggested that the delivery of Wnt pathway inhibitors to CSCs requires stable liposomal encapsulation and delayed tumor tissue drug release. Liposomal drugs specifically interfere with aberrant Wnt signaling in tumor tissues, resulting in a focused effect on LGR5^+^ CSCs (Li et al., 2019). Encapsulation in nanoparticles such as liposomes and nanomicelles has solved the problem of poor water solubility and bioavailability of natural products to be a favorable curcumin delivery system for targeting LC (Ibrahim et al., 2018; Zhang et al., 2018). Co-delivery of erlotinib and curcumin by nanomicelles improved anti-tumor effects *in vitro* by reducing Wnt pathway-related gene expression (Bagherian et al., 2021). Liposomes, as the DDSs we are concerned about, have been developed from the initial modification of classical lipid components to long-circulating, environmentally-sensitive and actively-targeted liposomes. It is expected that multifunctional liposomes with high potential for clinical applications can be obtained through organic combinations (Wang L. et al., 2021).

Nanoparticles can be modified to allow delivery of drugs by inhalation, which offers a promising non-invasive therapeutic option for the treatment of LC, such as a nanocomposite system loaded with RES for nebulization, demonstrating higher anti-cancer selectivity index (Wang et al., 2020; Zhang X. et al., 2023). Nanocrystalline materials have good atomization properties and are good carriers for local targeted delivery of natural products with poor water solubility to achieve continuous lung delivery (Lai et al., 2019). Other types of nanocarriers, such as chitosan nanoparticles, mesoporous silica nanoparticles, polymer nanoparticles, *etc.*, have also shown non-negligible development potential, and they not only improve bioavailability *in vivo*, but also enhance the synergistic delivery efficiency of chemotherapeutic agents (Liang et al., 2021). Quercetin and doxorubicin co-delivery using mesoporous silica nanoparticles decreased expression of Wnt16 and P-glycoprotein, thereby reshaping the tumor microenvironment and reversing multidrug resistance (Fang et al., 2018). Nano-delivery systems combining physical, biological and chemical means of targeting have shown promising anti-tumor efficacy and provide templates for multi-technology co-design of DDSs (Lin et al., 2020). However, various DDSs loaded with natural products still need to be explored in-depth preclinical experiments and clinical trials to clarify their safety and efficacy.

## 6 Summary

Current Wnt studies in LC have focused on LUAD-dominated NSCLC, and markers suggesting efficacy remain inadequately studied, although there have been reports on Wnt ligand-dependent tumor markers (Kleeman et al., 2020). In the future, more attention should be paid to the specific activation modes of Wnt in the LC, such as Wnt effector overexpression and inhibition by Wnt antagonists, which are common alterations in the LC. A fuller understanding of the possible LC-specific mechanisms of Wnt activation and analysis of the conformational relationships between natural product interventions and Wnt will facilitate research on more targeted inhibitors that may reduce the impact of widespread inhibition on normal human function.

Targeted toxicity of existing Wnt inhibitor therapeutic-grade molecules is a major limitation hindering their clinical development (Diamond et al., 2020; Angers, 2021). Compared with chemically synthesized medications, small-molecule compounds of natural product origin are less toxic, more biocompatible, and may present better safety and tolerability in humans. Among them, eugenol was observed to regulate the phosphorylation pattern of β-catenin in tumor cells to near normal, and no excessive inhibition or adverse toxic side effects were observed, indicating its potential as a novel regulator of Wnt (Choudhury et al., 2021). We are interested in this particular way of regulating the Wnt pathway, although we do not have a clear understanding of the constitutive relationship and the deeper mechanism of action of its intervention in the Wnt pathway because of the limited evidence of previous studies. Therefore, further conformational and experimental studies are required to investigate the mechanism of action and clinical therapeutic effects.

Although the limited number of studies and the incomplete study of Wnt inhibition modalities may have influenced us to draw definite conclusions, we found from the relevant studies that different types of compounds seem to play their own roles in interfering with the Wnt pathway in LC. Polyphenolic compounds, such as curcumin, have an excellent ability to inhibit oxidative stress-mediated upregulation of the abnormal Wnt pathway, an ability not possessed by other compounds. Half of the studied alkaloids and their derivatives could promote the stabilization of β-catenin destructive complex by up-regulating Axin, GSK-3β or downregulating its inactive phosphorylation. Terpenoids appear to act upstream of the Wnt pathway to inhibit it, and most terpenoids and derivatives, such as triptolide, inhibit the Wnt pathway in LC through the upregulation of Wnt antagonists and the downregulation of Wnt ligands. Natural small-molecule products have greatly increased the diversity and potential for drug development, with diverse mechanisms of action, thus allowing them to act on multiple targets of the Wnt pathway in LC and present partial inhibition of some of the more difficult targets, such as c-Myc, which may reduce the risk of future drug resistance.

In the context of the limited clinical studies on existing Wnt inhibitors, our study provides pharmaceutical companies and researchers with safer and more diverse options for the future development of inhibitors targeting the key Wnt pathway in LC, thereby enriching the clinical treatment options for LC.

## 7 Outlook

The complexity of the molecular interactions of the Wnt/β-catenin pathway and its involvement in different physiological functions make drug discovery challenging (Chatterjee et al., 2022). The introduction of new technologies such as artificial intelligence using, natural language processing, deep learning, and image recognition has greatly improved the efficiency and success rate of drug discovery (Freedman, 2019). Although the development of phytochemicals high-value of is in the spotlight, natural compounds isolated from living organisms currently face difficulties in screening new drugs due to their poor solubility, stability, and pharmacokinetics (Süntar et al., 2021). Nanoparticles and liposomes are useful strategies to overcome the poor absorption, rapid metabolism, and elimination inherent in most natural products; helping to increase their bioavailability and target specific sites, such as the lung. Given the emerging treatment landscape of non-invasive therapy in LC, a precise targeted local therapy should be attempted to reduce the systemic side effects of Wnt inhibitors.

In addition, the direct tumor-killing effect of Wnt inhibitors alone is limited. We are particularly interested in the impact of Wnt in mediating clinically relevant therapies for LC, such as chemotherapy, radiotherapy, EGFR-TKI, immunotherapy, and even antiangiogenic therapy. Therefore, Wnt combination therapy is a good strategy for overcoming drug resistance in LC, and should be an overriding direction for future research. Many naturally occurring small molecules used as Wnt inhibitors have good effects when used in combination with chemotherapy and targeted drugs, with the advantage of inhibiting proliferation and overcoming drug resistance, but the effectiveness and safety of clinical treatment still need to be further determined (Guo et al., 2021; Shah et al., 2021; Tian et al., 2021). The therapeutic treatment of LC could be significantly improved by the discovery of more effective and selective inhibitors of Wnt/β-catenin in combination with other therapies.

## Abbreviations

KRAS, Kirsten Rat Sarcoma Viral Oncogene Homologue; RAS, rat sarcoma viral oncogene homolog; FDA, The US Food and Drug Administration; Stat3, signal transducer and activator of transcription 3; CDKN1B, Cyclin‐dependent kinase inhibitor 1B; N-cadherin, neural-cadherin; E-cadherin, epithelial-cadherin; ZEB1, zinc-finger E-box binding homeobox 1; ZEB2, zinc-finger E-box binding homeobox 2; HOXB9, homeoboxB9; Oct4, octamer-binding transcription factor 4; BCL-XL, B-cell lymphoma-extra large; ALDH1, aldehyde dehydrogenase 1; BCL2L2, B cell lymphoma-2-like 2; BCL2, B cell lymphoma 2; IL-6, interleukin-6; Src, proto-oncogene tyrosine-protein kinase SRC; FAK, focal adhesion kinase 1; ERK, extracellular signal regulated kinase; HSP90, heat shock protein 90; KK1, Dickkopf-1; p70S6K, 70 kDa ribosomal protein S6 kinase.
